# Comparison of Serum HBsAg Quantitation by Four Immunoassays, and Relationships of HBsAg Level with HBV Replication and HBV Genotypes

**DOI:** 10.1371/journal.pone.0032143

**Published:** 2012-03-05

**Authors:** Edouard Tuaillon, Anne-Marie Mondain, Nicolas Nagot, Laure Ottomani, Dramane Kania, Erika Nogue, Pierre-Alain Rubbo, Georges-Philippe Pageaux, Philippe Van de Perre, Jacques Ducos

**Affiliations:** 1 Université de Montpellier 1, INSERM Unité 1058, Montpellier, France; 2 Départment de Bactériologie-Virologie, CHRU de Montpellier, Montpellier, France; 3 Institut de Recherche en Biothérapie, CHRU de Montpellier, Montpellier, France; 4 Départment de l'Information Médicale, Montpellier, France; 5 Laboratoire de Virologie, Centre Muraz, Bobo-Dioulasso, Burkina-Faso; 6 Département d'Hépato-Gastro-Entérologie et INSERM Unité 632, CHRU de Montpellier, Montpellier, France; Saint Louis University, United States of America

## Abstract

**Background:**

The decline in hepatitis B virus surface antigen (HBsAg) may be an early predictor of the viral efficacy of Hepatitis B virus (HBV) therapy. The HBsAg levels obtained by different immunoassays now need comparing and the relationships between levels of HBsAg and HBV DNA alongside HBsAg and genotype must be evaluated.

**Methodology/Principal Findings:**

HBsAg levels were compared among 80 patients using the Abbott Architect assay, a commercial immunoassay approved for HBsAg detection and quantitation, and three other assays derived from immunoassays approved for HBsAg detection (manufactured by Diasorin, Bio-Rad and Roche). Good correlation was found between the Abbot vs. Diasorin, Bio-Rad and Roche assays with narrow 95% limits of agreement and small mean differences: −0.06 to 0.11, −0.09 log_10_ IU/mL; −0.57 to 0.64, −0.04 log_10_ IU/mL; −0.09 to 0.45, −0.27 log_10_ IU/mL, respectively. These agreements were not affected by genotypes A or D. HBsAg was weakly correlated with HBV DNA, whatever the HBsAg assay used: Abbott, *ρ* = 0.36 p = 0.001, Diasorin *ρ* = 0.34, p = 0.002; Bio-Rad *ρ* = 0.37, p<0.001; or Roche *ρ* = 0.41, p<0.001. This relationship between levels of HBsAg and HBV DNA seemed to depend on genotypes. Whereas HBsAg (Abbott assay) tended to correlate with HBV DNA for genotype A (*ρ* = 0.44, p = 0.02), no such correlation was significant for genotypes D (*ρ* = 0.29, p = 0.15).

**Conclusion/Significance:**

The quantitation of HBsAg in routine clinical samples is comparable between the reference assay and the adapted assays with acceptable accuracy limits, low levels of variability and minimum discrepancy. While HBsAg quantitation is not affected by HBV genotype, the observed association between levels of HBsAg and HBV DNA seems genotype dependent.

## Introduction

There has been recent renewed interest in measuring serum levels of HBsAg as a surrogate marker to predict HBsAg loss and monitor anti-HBV therapy. During the natural history of HBV infection, the loss of serum HBsAg is generally associated with their seroconversion to anti-HBs, the hallmark of a successful immunological response to HBV infection. The development of therapies based on nucleotides and interferon in the last ten years has dramatically improved the survival of hepatitis B virus (HBV) infected individuals. While nucleotide analogue treatment is constrained by the need for extended therapy, and peg-interferon by a limited response rate, both can achieve a sustainable control of HBV replication [1], [2]. HBV therapy can induce a complete loss of HBsAg thus indicating the control of chronic infection in some patients. Indeed, HBsAg clearance has been reported in 11% of HBeAg positive patients and in 8% of HBeAg negative patients after 48 weeks of peg-interferon therapy [3]. Likewise, HBsAg clearance was reported in 3% of HBeAg positive patients after one year of treatment with lamivudine [4] and in 5% after 5 years treatment with adevovir [5]. These results should be compared with the 0.5 to 1% annual frequency of HBsAg clearance natural occurring in untreated HBV chronic infection [6], [7], [8], [9], [10]. Some reports have suggested that the baseline HBsAg titer may reflect the HBV reservoir as represented by intrahepatic covalently closed circular (ccc) DNA [11], [12], [13], [14], [15]. However, other studies failed to confirm this association or found a positive correlation only in patients with detectable HBeAg [16], [17].

The measure of HBsAg concentration in addition to HBV DNA and HBeAg may be a useful biological parameter to monitor HBV infection in patients treated with peg-interferon and nucleotide analogues [18], [19], [20], [21]. The speed and amplitude of the decline in HBsAg levels are suspected to be a good predictors of sustained virological response and HBsAg loss [3], [20]. Indeed, a decline in HBsAg levels of 0.5 and 1 log_10_ IU/mL 12 and 24 weeks after peg-interferon initiation, respectively, has been proposed to predict a sustained virological response [2], [3]. Alternatively, reaching HBsAg levels below 1500 IU/mL after 12 weeks [20] or below 10 IU/mL at the end of the treatment would also be expected to predict a sustained response [19]. Using a cutoff at 1000 IU/ml, HBsAg quantitation in combination with HBV DNA load may also be able to identify infection likely to remain inactive [22]. New algorithms including both HBsAg and HBV DNA serum levels should be assessed with the aim of optimizing treatment strategies. Whatever the thresholds selected, any upcoming therapeutic recommendations based on HBsAg level would impose a concordance among different assays concerning the measurement of anti-HBs.

The aims of the present study were to assess the performance of different methods to quantitate HBsAg. We sought here to demonstrate the potential utility of slightly modified qualitative assays to accurately measure HBsAg concentration in comparison with the Abbott Architect assay, a commercial quantitative assay. We also investigated the relationships between serum levels of HBV DNA and HBsAg, and how HBV genotype could impact this relationship and/or assay performance.

## Results

### Comparison of the values for HBsAg concentration between assays

All clinical specimens provided valid and quantifiable results for HBsAg level using the four assays. The 1/1000e dilution of the clinical samples allowed the measurement of a value within the range of quantitation in 92% of cases using the Abbott assay; in 79% using the Diasorin assay; 76% using Bio-Rad, and 96% using Roche. Additional lower (undiluted or 1/20e) or higher (1/10000e) dilutions were required for the remaining clinical samples.

The median (IQR) HBsAg concentrations were 3.68 (4.13-3.27) log_10_ IU/mL with the reference Abbott assay; 3.79 (4.18-3.36) log_10_ IU/mL with Diasorin; 3.75 (4.16-3.18) log_10_IU/mL with Bio-Rad; and 3.49 (3.79-3.01) log_10_ IU/mL with Roche.

**Figure 1:**
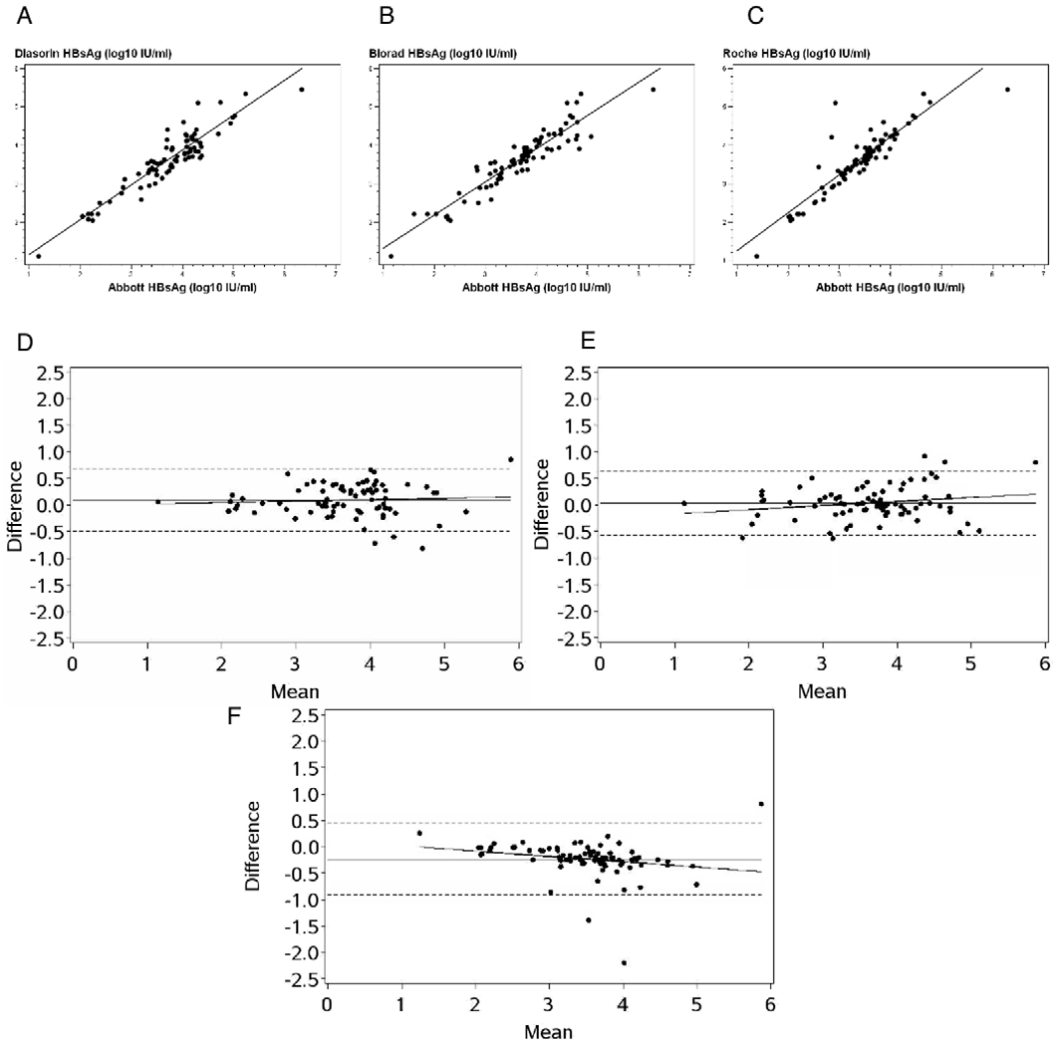
Comparison of the AgHBs concentrations between Abbott and Diasorin, Bio-Rad, Roche assays. Linear regression analysis of HBsAg levels (expressed in log_10_ IU/ml) obtained by (A) Abbott vs. Diasorin, (B) Abbott vs. Bio-Rad, Abbott vs. Roche immunoassays, and Bland-Altman plot of HBsAg level differences between (D) Abbott and Diasorin, (E, F) Abbott and Bio-Rad, (G) Abbott and Roche immunoassays. The horizontal solid lines indicate the mean titer difference values, the dashed lines represent the ±1.96-SD limits from the means, and diagonal solid lines are the linear regression lines.

The Diasorin assay correlated well with the reference assay, *ρ* = 0.88 (p<0.001) (Fig. 1.A). The difference between 7 (9%) paired results was above 0.5 log_10_IU/mL. The concordance plot (Fig. 1.D) confirmed the strong correlation with the Abbott method with narrow 95% limits of agreement (−0.5 to 0.68) and a weak difference mean of 0.09 log_10_ IU/mL.

A strong correlation was observed between Bio-Rad and Abbott methods; *ρ* = 0.93 (p<0.001) (Fig. 1.B). Ten values for HBsAg concentration (13%) estimated by the Bio-Rad method differed from the Abbot results by more than 0.5 log_10_IU/mL. According to the Bland-Altman method, the mean difference was 0.04 log_10_ IU/mL, and narrow limits of agreement (−0.57 to 0.65) were achieved (Fig. 1.E).

Results obtained with the Roche assay were also highly correlated with the Abbott assay, *ρ* = 0.88 (p<0.001) (Fig. 1.C). A difference between the two test values was above 0.5 log_10_ IU/mL in 8 (10%) of the paired results. The Bland-Altman approach (Fig. 1.F) showed a good agreement with a mean difference of −0.23 log_10_ IU/mL (limits of agreement: −0.9 to 0.45). There was no proportional bias for the three assays compared to the reference assay.

Similar comparisons were performed between the three modified assays (data not shown). HBsAg quantitation obtained with Diasorin vs. Roche assays presented acceptable mean differences (0.32 log_10_ IU/mL,) and strong correlation (*ρ* = 0.82, p<0.0001), likewise for Bio-Rad versus Roche (mean differences of 0.27 log_10_ IU/mL and *ρ* = 0.91, p<0.0001) and Diasorin vs. Bio-Rad (mean differences of −0.05 log_10_ IU/mL and *ρ* = 0.82, p<0.0001).

### Impact of HBeAg status on HBsAg level

The median (IQR) of the log_10_ IU/mL HBsAg concentration was higher in the 19 patients with detectable HBeAg than in the 61 patients with undetectable HBeAg (data not shown), 4.14 (3.45–4.78) vs. 3.62 (3.12–3.91) using the Abbott assay (p = 0.03); 4.27 (3.69–4.74) vs. 3.73 (3.32–4.08) with the Diasorin (p = 0.001); 4.06 (3.55–4.65) vs. 3.71 (3.11–3.98) with the Bio-Rad (p = 0.031), and 3.54 (3.08–4.43) vs. 3.49 (2.99–3.68) with the Roche (p = 0.089). Similarly, the median (IQR) HBV DNA levels tended to be higher among individuals with detectable HBeAg 4.54 (3.17–6.0) log_10_ IU/mL vs. 3.44 (2.73–4.79) log_10_ IU/mL (p = 0.059), data not shown).

### Relationship between levels of HBV DNA and HBsAg

**Figure 2:**
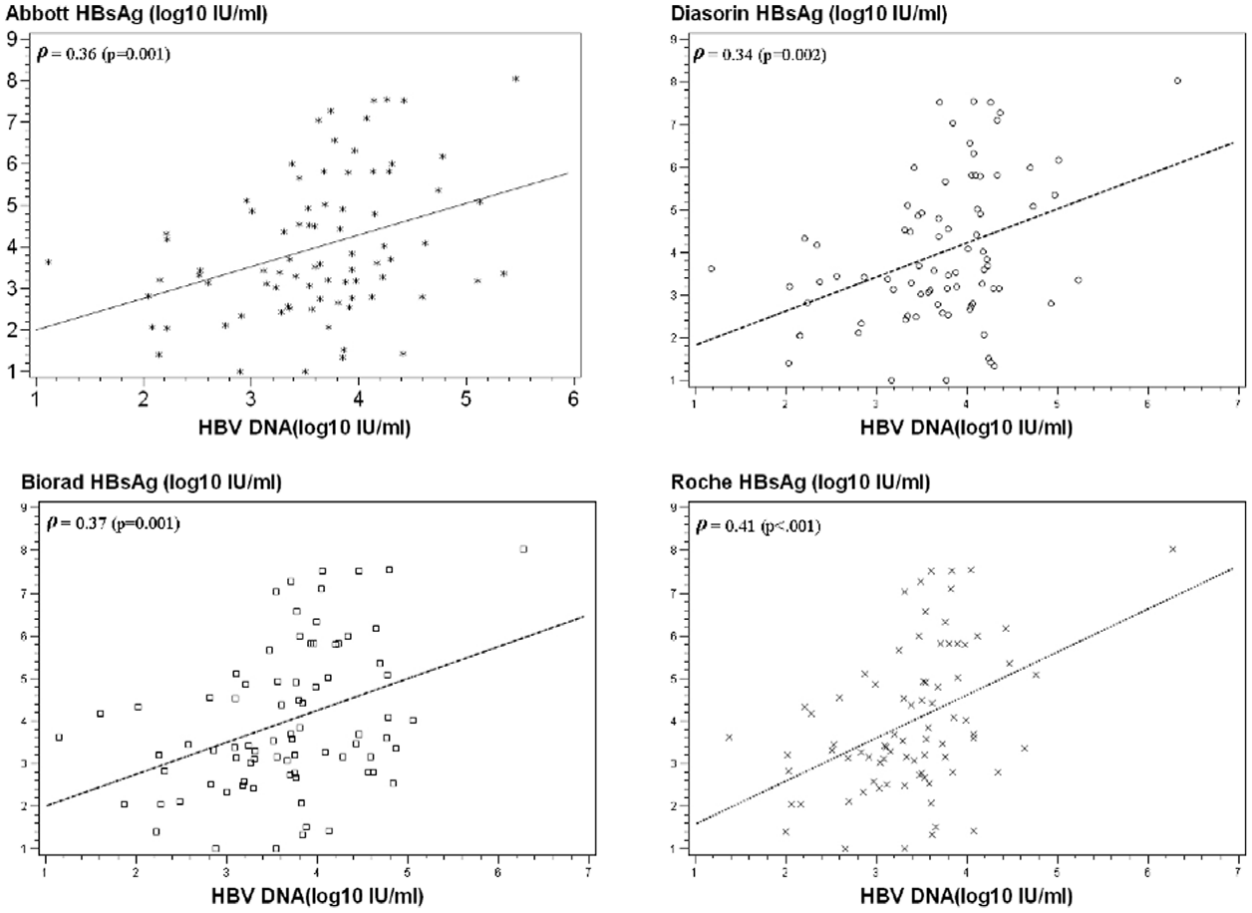
Correlation of HBsAg (log IU/mL) and HBV DNA (log IU/mL). (A) Abbott, (B) Diasorin, (C) Bio-Rad, (D) Roche immunoassays.

Overall, levels of HBsAg and HBV DNA were weakly correlated, whatever the HBsAg assay used (Fig. 2). Taking into account the HBeAg status (+/−), the correlations between HBsAg and HBV DNA levels remained weak, with an identical correlation for HBeAg-negative patients and HBeAg positive patients of 0.33 using the Abbott assay (data not shown).

**Figure 3:**
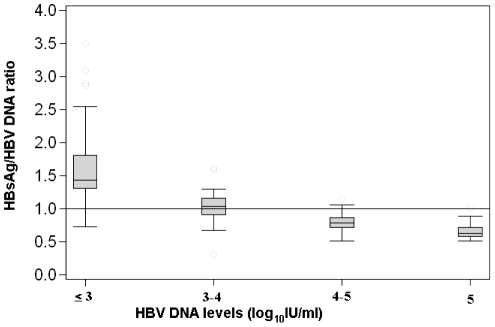
Plots of Log_10_ IU/ml HBV-DNA/HBsAg ratio by levels of HBV replication.

The HBsAg (log_10_ IU/mL, Abbott assay) to HBV DNA (log_10_ IU/mL) ratio was assessed on serum samples stratified on HBV DNA level (≤3 log_10_ IU/mL, 3–4 log_10_ IU/mL, 4–5 log_10_ IU/mL, and >5 log_10_ IU/mL). The median ratio was higher in specimens with low HBV DNA values than with high HBV DNA values (p<0.001) (Fig. 3), median (IQR): 1.42 (1.30; 1.80); 1.03 (0.91; 1.16); 0.78 (0.71; 0.86), 0.63 (0.57; 0.72) for HBV DNA levels ≤3 log_10_ IU/mL, 3–4 log_10_ IU/mL, 4–5 log_10_ IU/mL, and >5 log_10_ IU/mL, respectively. These results suggest that the secretion of HBsAg is more highly conserved than the HBV DNA production among patients in whom viral replication has been brought under control by the host.

**Figure 4:**
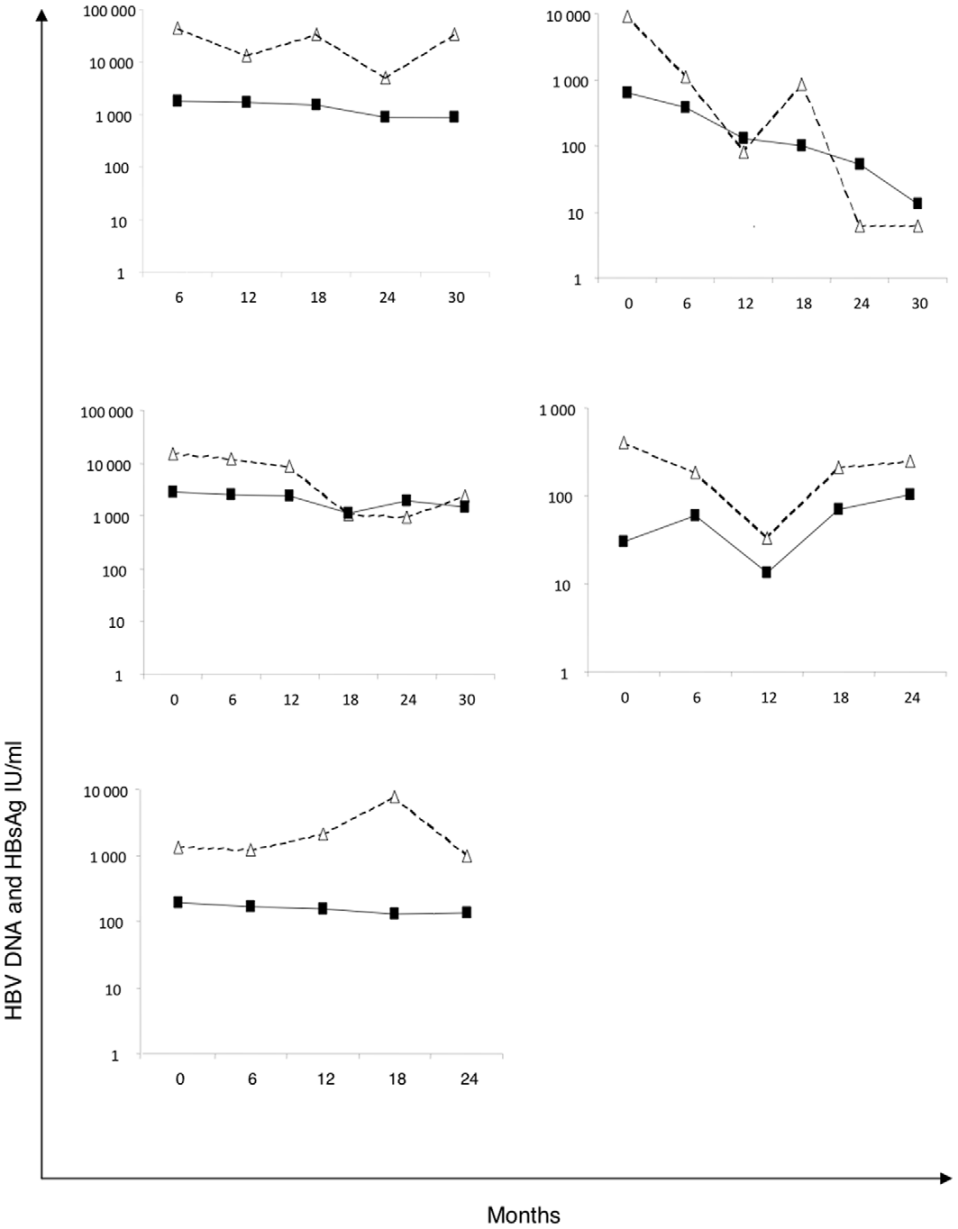
Temporal evolution of HBsAg and HBV DNA in five patients with persistente HBV infection. HBsAg log_10_ IU/ml (▪ and solid line), HBV DNA (▵ and dotted line).

We then analyzed the temporal evolution of the levels of HBsAg levels measured by the Abbott assay in parallel with those of HBV DNA in a longitudinal study of five patients with persistent HBV infection. None of the patients had received anti-HBV drugs and they were all negative for HBeAg. Twenty-two samples were explored over the 2-year follow-up period. We observed a low variation of HBsAg on sequential testing: the mean (±SD) variation between sequential HBsAg quantitative measurements was 16% (±98) (Fig. 4). By contrast, the mean (±SD) variation of HBV DNA between sequential measurements was 138% (±322). Fluctuations in levels of HBsAg were generally accompanied by those of HBV DNA.

### Impact of HBV genotype on measured levels of HBsAg

**Figure 5:**
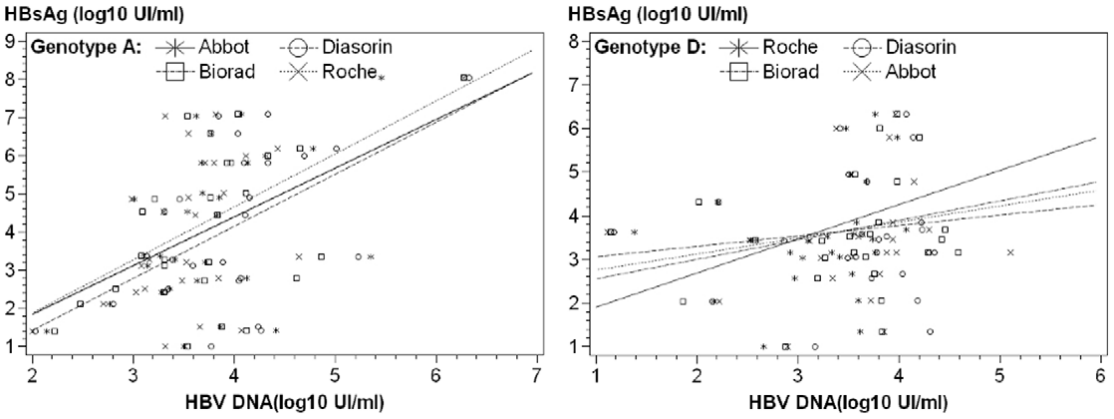
Correlation of HBsAg (log_10_ IU/mL) and HBV DNA (log_10_ IU/mL). (A) VHB genotype A, Abbott *ρ* = 0.44, p = 0.02; Diasorin *ρ* = 0.49, p = 0.01; Bio-Rad *ρ* = 0.46, p = 0.02; Roche *ρ* = 0.47, p = 0.02; (B) genotype D, Abbott *ρ* = 0.29, p = 0.15; Diasorin *ρ* = 0.05, p = 0.81; Bio-Rad *ρ* = 0.30, p = 0.14; Roche *ρ* = 0.38, p = 0.05.

The HBV genotyping was available for 69 patients. Twenty seven subjects were infected by HBV genotype A, 5 by genotype B, 1 by genotype C, 26 by genotype D, 8 by genotype E and 2 by genotype G. The clinical features and basic biological features (ALT, AST, and HBV DNA) were similar among the three main HBV-genotypes (Table 1). Similar HBsAg concentrations were obtained by the Abbott method and the three other assays for the genotypes A, and D (data not shown). For genotype A, the median concentrations (log_10_ IU/mL) were: Abbott, 3.69 (3.36; 4.12); Diasorin, 4.04 (3.39; 4.26); Bio-Rad, 3.77 (3.30; 4.12), and Roche, 3.54 (3.11; 3.84). For genotype D they were: Abbott, 3.68 (3.23; 3.94); Diasorin, 3.76 (3.41; 4.19); Bio-Rad, 3.74 (3.24; 3.99); and Roche, 3.50 (2.97; 3.68). For Thus the concordance between the assays appeared unaffected by the genotypes A and D. Likewise, the HBsAg levels were comparable among genotypes for each method (p_Abbot_ = 0.58, p_Diasorin_ = 0.25, p_Bio-Rad_ = 0.59, p_Roche_ = 0.20), as were the medians of the HBsAg/HBV-DNA ratio (data not shown). By contrast, the relationship between HBsAg and HBV DNA levels did appear to be genotype dependent. We observed a correlation between HBsAg and HBV DNA levels for genotype A (*ρ* = 0.44, p = 0.02) using the Abbot assay but not for genotypes D (*ρ* = 0.29, p = 0.15). The same observation was made using the three adapted assays (Fig. 5).

**Table 1:** Clinical and basic features for the two main genotypes.

	Genotype A (n = 27)	Genotype D (n = 26)	Kruskall Wallis
	Median (IQR)	Median (IQR)	P value
**ALT** (IU/L)	**29.50** (22.00–43.00)	31.00 (25.00–90.00)	0.37
**AST** (IU/L)	**31.50** (25.00–68.00)	34.00 (21.00–44.00)	0.68
**HBV DNA** (log_10_ IU/mL)	**3.37** (2.53–5.82)	**3.45** (3.03–4.32)	0.60
**HBsAg Abbott** (log_10_ IU/mL)	**3.69** (3.36–4.12)	**3.68** (3.23–3.94)	0.57
**HBsAg Diasorin** (log_10_ IU/mL)	**4.04** (3.39–4.26)	**3.76** (3.41–4.19)	0.25
**HBsAg Bio-Rad** (log_10_ IU/mL)	**3.77** (3.30–4.12)	**3.74** (3.24–3.99)	0.59
**HBsAg Roche** (log_10_ IU/mL)	**3.54** (3.11–3.84)	**3.50** (2.97–3.68)	0.41

Results for genotypes B (n = 3), C(n = 1), E (n = 8) and G (n = 2) are not presented.

## Discussion

Among the several assays quantifying HBsAg that are commercially available in Europe - Elecsys II (Roche Diagnostics), ADVIA Centaur HBsAg assay (Bayer), Hepanostika HBsAg (Biomerieux) [23] - the Architect assay has been the most frequently cited in published studies. Here, the Architect HBsAg test currently approved to monitor HBsAg concentrations in clinical practice was used as the reference assay. This assay is based on a chemiluminescent reaction and offers the advantage of automated quantitation and standardization. On the other hand the Architect assay is a single-use test format that requires dedicated equipment using an advanced technology. While the HBV pandemic has affected countries worldwide, the burden of this infection is most felt in resource-limited countries. Therefore, pressure to lower the cost of HBV antiviral therapy, including not only the drugs against HBV but also the assays used for monitoring infected patients is a continuous challenge. Enzyme immunoassays are less expensive than the DNA assays used for the genomic quantitation of HBV in serum. In addition, assays using colorimetric detection are less technically demanding than chemiluminescent immunoassays with respect to required facilities and instrumentation. Finally, most of these tests use a microplate format and may be performed manually.

In our study, the quantitation of HBsAg levels in routine clinical samples by different test systems appeared overall to be accurate, showing low variability and little discrepancy when compared with the reference assay. Thus the HBsAg level appears as a robust biological parameter that can be accurately measured by different ELISA assays. While most enzyme immunoassays devoted to HBsAg detection are not approved for HBsAg quantitation, they may be used for this purpose. The lower limit of quantitation was found to be close to cutoffs obtained with the chemiluminescent immunoassays and was adequate to efficiently quantify all serum samples tested in our study. In addition, this lower limit of quantitation is markedly below the clinically significant threshold of 10 IU/mL proposed by Brunetto et al. [19]. Although the colorimetric assays used in our study require the establishment of a calibrated curve, they do offer several advantages over the Architect test, notably, they are simple, inexpensive, and have analytical performance characteristics similar to those obtained with the commercial HBsAg quantitative assay. Nevertheless, the differences observed between assays in the Bland-Altman plots suggest that the same assay should be used throughout the whole period of therapeutic monitoring for any one patient.

The measurement of HBsAg levels would be particularly well adapted to the treatment of HBeAg negative patients. HBeAg-negative hepatitis variants with mutations in the precore or basic core promoter regions are frequent among chronic HBV carriers in Mediterranean countries, and represent the majority of chronically infected patients in our hospital (89%, personal data). HBsAg levels are lower in HBeAg negative than in HBeAg positive samples, as previously reported [24]. This observation is in line with a previous report indicating that pre-S deletion involved in HBeAg negative HBV infection decreases the synthesis of small surface antigens [25].

In the current transversal study patients were explored in different phases of HBV infection without stratification in the various clinical presentation of chronic hepatitis B. This limits the possibilities to fully characterize the complex association of HBsAg and HBV DNA levels during the natural history of chronic hepatitis B. However, our results indicated that the poor correlation reported between HBsAg and HBV DNA levels [11], [19], [20] is not related to the test used. In addition, using repeated measures of HBsAg and HBV DNA levels, we observed stable HBsAg concentration in the absence of significant HBV DNA and ALT variations. Previous studies have reported that fluctuations in HBsAg level are associated with changes in HBV DNA level [11], [26].The highest correlations between HBV DNA and HBsAg levels are found in early phases of infection, during acute infection, immune clearance and HBeAg positive hepatitis B persistent infection [17], [27], [28]. In advanced phases of infection, the level of replication is generally low and associated with the production of much more defective viral HBsAg particles secreted as noninfectious filamentous or spherical subviral particles [25], [30]. Hence, in later phases of infection when the ratio of HBsAg/HBV-DNA is low, the association between HBsAg production and HBV DNA replication seems weakest or “disconnected” [19].

Our results from all four assays used in this study demonstrated that there is no impact of HBV genotype on HBsAg quantitation or on the ratio of HBsAg/HBV DNA. However, the efficiency of the HBV DNA template to produce HBsAg may be differently affected by HBV genotype diversity. Indeed, we observed a weak correlation between HBsAg production and HBV DNA replication for genotype A but not for genotype D. Based on *in vitro* observations of HBV DNA replication, genotype A virus appears to have a lower replication rate by comparison with other genotypes [29]. However, *in vivo*, higher HBsAg levels have been reported at baseline for HBV genotypes A and D by comparison with other genotypes [19]. By contrast with our results and using the same PCR method, Wursthom et al. [21] recently reported a correlation between HBsAg and HBV DNA for genotype D, but not for genotype A. Such discrepancy between studies could be explained at least partly by clinical difference in the population of patients that we did not take into account. Further studies are required to better characterize the association of HBsAg serum levels with the various clinical settings of chronic HBV infection.

In conclusion, the quantity of HBsAg can be efficiently measured using commercially available assays developed for qualitative assessment. These methods are appropriate for the monitoring of patient response to antiviral therapy. Some highly cost-effective assays, such as colorimetric assays, are applicable in resource-constrained countries. To ensure a better use of HBsAg quantitation during therapy, studies are needed to establish clinically relevant thresholds according to HBV genotype, HBeAg status, HBsAg/HBV DNA ratio and clinical stage of HBV persistent infection.

## Materials and Methods

### Patients

The study included 80 adult patients chronically infected by HBV and untreated for HBV infection at the time of sampling. The study was approved by the institutional ethics research committee and patients provided written informed consent. Subjects included were 51 men and 29 women with a median age of 44 years. The median (interquartile range-IQR) HBV DNA serum level was 3570 (628-114500) IU/mL. The serum concentrations of alanine amino transferase (ALT) and aspartate aminotransferase (AST) were 31 (21–62) IU/L and 31 (22–50.5) IU/L respectively. Nineteen patients (24%) were HBeAg positive. Among HBeAg negative patients, twenty one (26%) had a status of inactive carrier with persistently normal ALT and HBV DNA below 2000 IU/mL. Twenty seven clinical serum specimens collected from five patients included in the study were used for sequential HBV viral load and HBsAg testing over a two-year period. These patients were negative for HBeAg with persistently normal ALT and HBV DNA ranging from 594 to 664 000 IU/mL at baseline.

### Standard HBsAg preparation

The standard HBsAg consisted of a reference preparation of plasma purified HBsAg purchased from Biokit (Barcelona, Spain) that contained Ayw and Adw HBsAg in equal quantities. This standard preparation was serially diluted in a pool of HBsAg negative serum samples in order to obtain concentrations between 0.03 and 250 IU/mL. The dilutions of the reference HBsAg were used to investigate the range of linearity of the assays and to establish standard curves [30].

### Assay systems

Three different automated immunoassays were compared with the Architect HBsAg QT assay, a commercial quantitative assay developed by Abbott laboratories (Abbott, Chicago, IL).

- The Abbott assay is a chemiluminescent microparticle immunoassay approved in Europe for the detection and quantitation of HBsAg. It is internally calibrated using the World Health Organization standard for HBsAg [31], and measures HBsAg concentrations within the range of 0.05 to 250 IU/mL. The manufacturer recommends a 1∶500 dilution of the test samples. Samples with HBsAg levels above or below this range require a lower or greater dilution in the manufacturer's diluent to bring them into the range of the calibration curve. The lower limit of detection of this assay is 0.05 IU/mL.
- The ETI-MAK-4 assay (DiaSorin, Turin, Italy) is a conventional enzyme immunoassay using 96 well microplates. We performed this assay on a Triturus automate (Grifols, Barcelona, Spain). The lower limit of detection is 0.05 IU/mL and 0.03 PEI U/mL. Optical densities were reported on a standard curve to calculate the values expressed in IU/mL.
- The Monolisa HBs Ag ULTRA assay (Bio-Rad Laboratories, Redmond, WA) is another conventional enzyme immunoassay using 96 well microplates. This assay was run on an EVOLIS Twin Plus automate (Bio-Rad Laboratories). The lower limit of detection with this assay is 0.50 pg/mL. Optical densities were reported on a standard curve to obtain the results in IU/mL.
- The Roche HBsAg II assay on the Cobas e411 system is a chemiluminescent microparticle immunoassay (Roche Diagnostics GmbH, Mannheim, Germany) used for HBsAg detection. The lower limit of detection of this assay is 0.1 IU/mL and 0.1 PEI U/mL. The chemiluminescent reaction measured in relative light units was reported on a standard curve to calculate values expressed in IU/mL.

Patient samples preparations were diluted in a pool of decomplemented sera negative for HBsAg, anti-HBc and anti-HBs antibodies (1∶1000 dilution) to assess HBsAg concentration using the three modified assays. Samples with results falling beyond the linear range of the standard curve were retested in lower or greater dilutions. Apart from the dilution step, HBsAg analyses were performed in accordance with the manufacturer's recommendations.

### Calibration curves, ranges of quantitation and reproducibility

The linear range for HBsAg concentration measurements for the adapted Diasorin, Bio-Rad and Roche HBsAg quantitative assays was assessed using serial dilutions of the standard. The following quantitation ranges were retained: 0.24 to 62.5 IU/mL for Diasorin and Bio-Rad assays and 0.24 to 125 IU/mL for the Roche assay. A strong linear relationship between the optical density or relative light units and HBsAg concentration was observed in the three assays, (r^2^>0.999, data not shown). The lower limit of quantitation was set as the lowest point of the standard dilution used for the calibration curve. Replicate aliquots of the standard preparation (1.8 log_10_ IU/ml) were also used to evaluate the assay precision by calculating the percent coefficient of variation (%CV) for log_10_-transformed data over nine separate runs. Mean %CV were 9.6% for Abbott, 13.2% for Diasorin, 10.0% for Bio-Rad, and 4.2% for Roche assays (data not shown).

### HBV DNA viral load

HBV DNA viral load was measured using the CobasAmpliPrep/CobasTaqMan HBV Test, v2.0 (Roche Diagnostics GmbH, Mannheim, Germany). The HBV DNA quantitation range was 20 to 1.7.10^8^ IU/mL with the correlation coefficient of the standard curve consistently exceeding 0.990. In this assay, 4.86 copies/mL equaled 1 IU/mL.

### HBV genotyping

HBV genotyping was obtained for 35 patients. DNA was extracted from 200 µl of serum using the QIAamp® DNA Blood Mini Kit (Qiagen GmbH, Hilden) according to the manufacturer's protocol. HBV genotype was determined using the TRUGENE® HBV Genotyping Kit for use with the OpenGene® DNA Sequencing System (Siemens Healthcare Diagnostics Inc, Tarrytown, USA). The TRUGENE HBV Module 1.0 contains all the necessary software components to perform HBV genotyping analysis. The query sequence is in turn compared to the consensus sequences of genotypes A through to G in the TRUGENE HBV Module of the OpenGene system software in order to determine the HBV genotype of the sample (Mutations in both Sag and RT/Pol regions are also detected and reported).

### Statistical Analysis

A logarithmic transformation was applied to HBsAg and HBV DNA concentrations. Continuous variables were expressed as medians (IQR).

The agreement and relationship between the Abbott Architect assay and the three modified assays were studied using Bland-Altman Plots and Spearman's correlation coefficients.

The impact of HBeAg status on HBsAg and HBV DNA levels was analyzed using Wilcoxon Mann Whitney test.

The association between HBV DNA replication and HBsAg production was assessed using the Spearman's correlation coefficient, and by analyzing the ratio of HBsAg (log_10_ IU/mL), measured by the Architect assay, to HBV DNA (log_10_ IU/mL) with the Jonckheere-Terpstra test.

The impact of genotype on HBsAg and HCV DNA levels was assessed using a Wilcoxon Mann Whitney test.

Statistical tests were two-sided and a p-value≤0.05 was considered statistically significant. The statistical software SAS version 9.2 (SAS Institute, Cary, N.C.; proc npar1way, proc univariate) was used for the statistical analyses.
